# Supplementary material for: Ultramicronized N-Palmitoylethanolamine Regulates Mast Cell-Astrocyte Crosstalk: A New Potential Mechanism Underlying the Inhibition of Morphine Tolerance
Source: Biomolecules. 2023 Jan 25;13(2):233. doi: 10.3390/biom13020233 (PMC9953591; doi:10.3390/biom13020233)
Supplement: Supplementary file 1 [file biomolecules-13-00233-s001.zip › biomolecules-2106954-supplementary.pdf]

Supplementary Materials

Methods for Histamine Dosage

The chromatographic method is reported in Table S1.

Time (min.)	A (%)	B (%)	Flow A+B (mL min^{-1})	Flow C (mL min^{-1})	Valve Position
0.00	10	90	0.25	0.5	Load
2.00	10	90	0.25	0.5	Inject
9.00	80	20	0.25	0.5	
12.00	80	20	0.25	0.5	
12.01	10	90	0.25	0.5	Load
25.00	10	90	0.25	0.5	

Table S1: chromatographic gradient with valve position switch time.

Flow parameters were optimized considering the properties of the used columns and the characteristics of ESI source in order to obtain the best performances.
To better understand how the injection valve works in 2D-HPLC system, a scheme is reported in Figure S1.

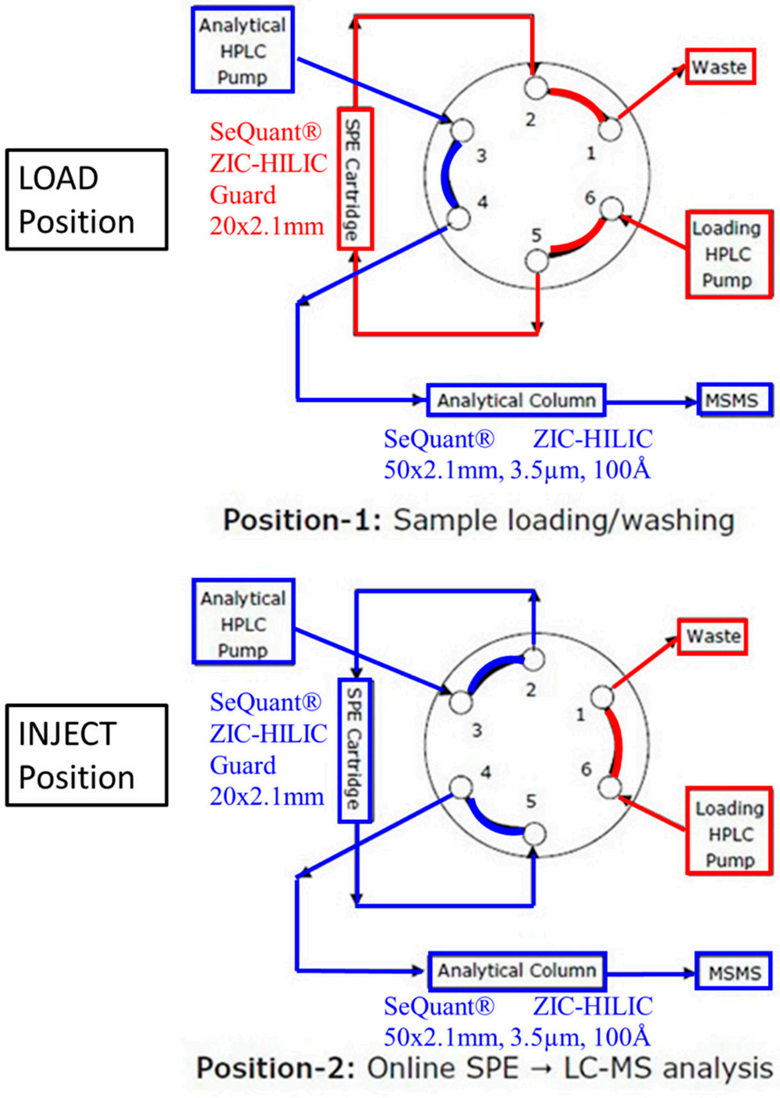

Figure S1: the injection valve position in 2D-HPLC system.

Injection volume was 50 μL.

The source used was an ESI source operating in positive ion and using the following settings: 5kV needle, 42 psi nebulizing gas, 600 V shield, and 20 psi drying gas at 280 °C. The energy resolved mass spectrometry (ERMS) experiments were performed to study the fragmentation of histamine and $[^2H_4]$-histamine and build their breakdown curves. The ERMS experiments were carried out by a series of product ion scan (MS/MS) analysis, increasing the collision voltage (CV) stepwise in the range 5–50 V. Each MS/MS spectra were acquired in the m/z range from 50 to 130, scan time of 600 ms and argon was used as collisional gas. The ERMS experiments were performed by introducing working solution 1 of each analyte, via syringe pump at 10 μL min^{-1}; the protonated molecule was isolated, and the abundance of product ions were monitored. The ERMS data were used to build the breakdown curves that describe the fragmentation of precursor ion in relation to the collision voltage applied. The breakdown curves were obtained by plotting the relative intensity values (averaging about 15–20 scans) of each signal present in the MS/MS spectra acquired for each CV. In order to increase the sensitivity and the selectivity it was decided to work in MRM. The MRM transitions selected for the ID/2D-HPLC-MS/MS method are reported in Table S2.

Name	Precursor Ion (m/z)	Quantifier Ion (m/z)	Qualifier Ion (m/z)
$[^2H_4]$-histamine (IS)	116.0	99.0	-
Histamine	112.0	95.0	68.0

Table S2: MRM parameters.

For the validation of the method it was taken inspiration from EMA and ICH Q2(R1) guidelines [112,113].
Stock solutions of histamine and $[^2H_4]$-histamine (internal standard or IS) were prepared in 10 mM HCOOH in mQ water at 1.0 mg mL^{-1} and stored at 4 °C. Working solutions of each analyte were freshly prepared by diluting stock solutions up to a concentration of 1.0 mg mL^{-1} and 0.1 mg mL^{-1} (working solutions 1 and 2, respectively) in mixture of mQ water:acetonitrile 50:50 (v/v). The IS working solution was prepared in acetonitrile at 1.0 mg mL^{-1} (IS solution). A six-level calibration curve was prepared by adding proper volumes of working solution (1 or 2) of each analyte to 10 mL of IS solution. The obtained solutions were dried under a gentle nitrogen stream and dissolved in 1.0 mL of 10 mM of formic acid in mQ water:acetonitrile 50:50 (v/v) solution. Final concentrations of calibration levels of each analyte were:0, 2.5, 5.0, 10.0, 25.0, 50.0, and 100.0 ng mL^{-1}. All calibration levels were analyzed six times by the 2D-HPLC-MS/MS method described. Each sample was prepared by adding at 100 L of culture medium 10uL of IS solution. Then it was added 500 L of 10 mM formic acid in acetonitrile doing a protein precipitation. The obtained solutions were dried under a gentle nitrogen stream and dissolved in 1.0 mL of 10 mM of formic acid in mQ water:acetonitrile 50:50 (v/v) solution. Each sample batch were included the blank culture medium, prepared as described above, but by adding only the IS solution. Thus, the analysis of the blank samples can check any interference in the analyte MRM signals due to the matrix components. Three sets of six replicates for each analyte were prepared to evaluate the matrix effect (ME) and the analyte recovery (RE) of the proposed method [114]. In order to estimate precision and accuracy of the methods, a new series of samples at three concentration levels (low, medium and high levels), corresponding to 25, 50 and 100 ng mL^{-1} respectively, were prepared in six replicates for each compound following the procedures described above from the stock solutions in human plasma matrix. All these samples were analyzed with the method described above. The selectivity was achieved by using $[^2H_4]$-histamine as internal standard, evaluating the retention time (Rt) for each sample and monitoring two product ions for histamine.
A possible carry-over effect was evaluated by analyzing a blank sample after injecting the highest concentrated calibration level. No carry-over effect was found.
LOD and LOQ were estimated by following the standard deviation of the response and slope suggested by the ICH guidelines. The obtained linear regressions coefficient, the determination coefficient (R^2) and the estimated Limit of Detection (LOD) values for each analyte are reported in Table S3.

Name	Slope (PAR/ng mL^{-1})	Intercept (PAR)	R^2	LOD (ng mL^{-1})	LOQ (ng mL^{-1})
Histamine	0.0968	0.1047	0.9968	4.4	13.2

Table S3: linear regression coefficients and LOD

Accuracy and precision were calculated by quantifying a series of specific samples as described before and the results are reported in Table S4.

Compound	Low level	Medium level	High level
	Recovery ± RSD (%)	Recovery ± RSD (%)	Recovery ± RSD (%)
Histamine	97 ± 5	101 ± 4	96 ± 4

Table S4: Accuracy & Precision(RSD) values for histamine

Finally, matrix effect and recovery were calculated by analyzing a specific batch of sample described before and by using the Matuszewski et al. protocol [114] (Table S5).

Name	ME (%)	RE (%)
Histamine	114	98

Table S5: ME & RE values for histamine

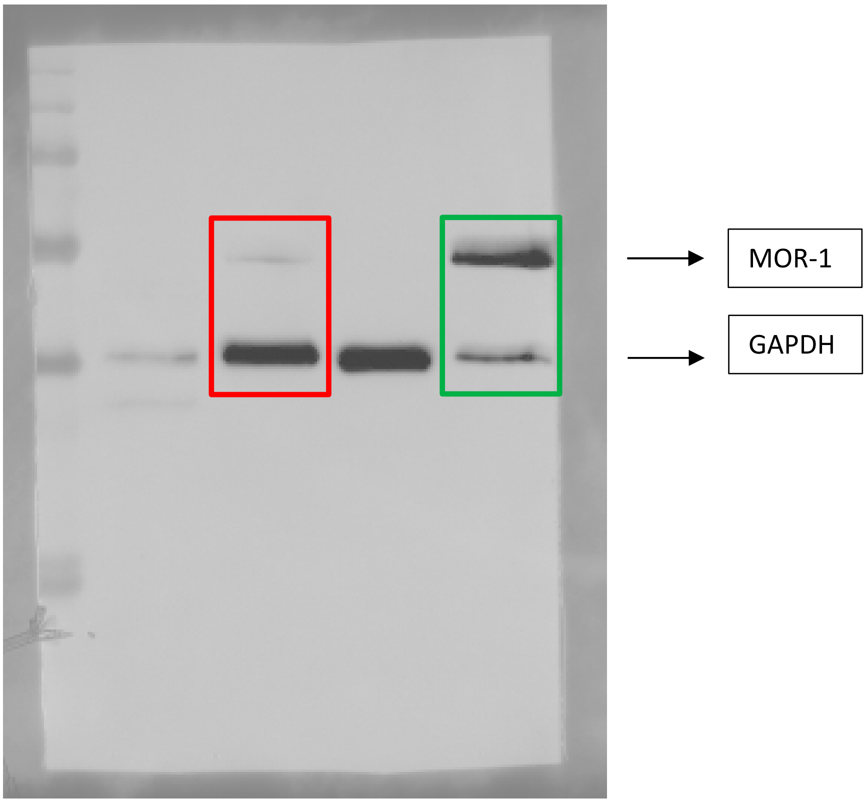

Figure S2. Entire Western Blot of MOR-1 in RBL-2H3. In the red box there are MOR-1 (μ-receptor) and GAPDH bands of RBL-2H3 lysate (in the green box there are MOR-1 and GAPDH bands of primary rat cortical astrocytes lysate).

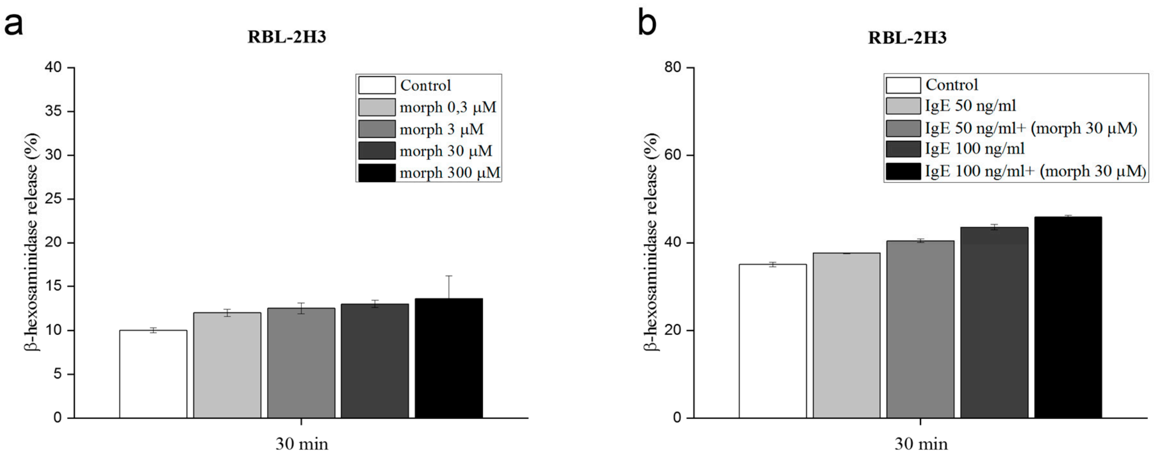

Figure S3. Optimization of RBL-2H3 degranulation protocol. (**a**) β-hexosaminidase assay of RBL-2H3 treated for 30 minutes with increasing concentrations of morphine (0.3-300 μM). (**b**) β-hexosaminidase assay of RBL-2H3 pre-treated with anti DNP-IgE (50-100 ng/mL) for 18 h and the day after with morphine 30 μM for 30 minutes. Values are expressed as the mean ± S.E.M. of three experiments.

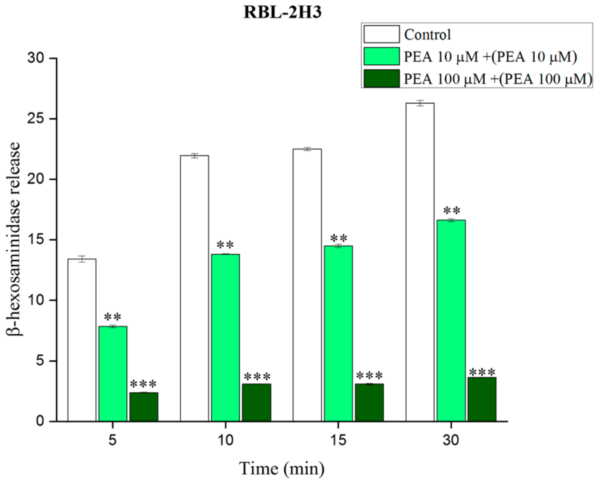

Figure S4. Effect of PEA 10 and 100 μM on RBL-2H3 degranulation. β-hexosaminidase assay of RBL-2H3 pre-treated for 18 h with PEA 10 and 100 μM and for further 30 minutes the following day, before being challenged with DNP-BSA for 5, 10, 15, 30 minutes. Values are expressed as the mean ± S.E.M. of three experiments. ** $p < 0.01$; *** $p < 0.001$ vs. control RBL-2H3.

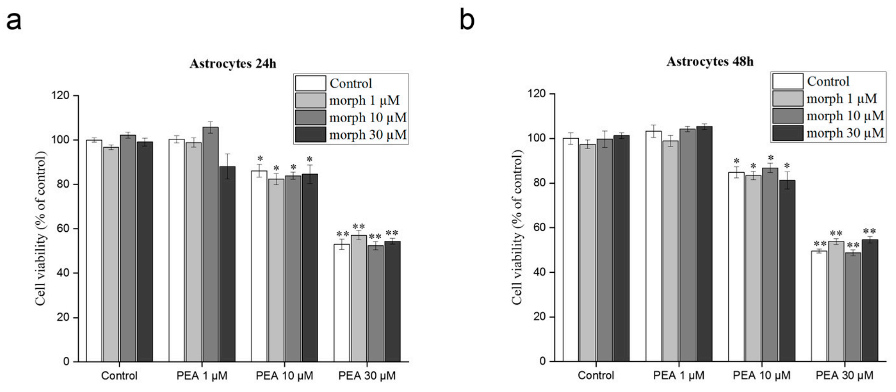

Figure S5. Effect of morphine and PEA on astrocyte viability. (**a-b**) MTT of rat primary cortical astrocytes treated with increased concentrations of PEA (1-30 μM), alone or in combination with increasing concentrations of morphine (1-30 μM) for 24 and 48 h. Values are expressed as the mean ± S.E.M. of three experiments. * $p < 0.05$; ** $p < 0.01$ vs. control astrocytes.

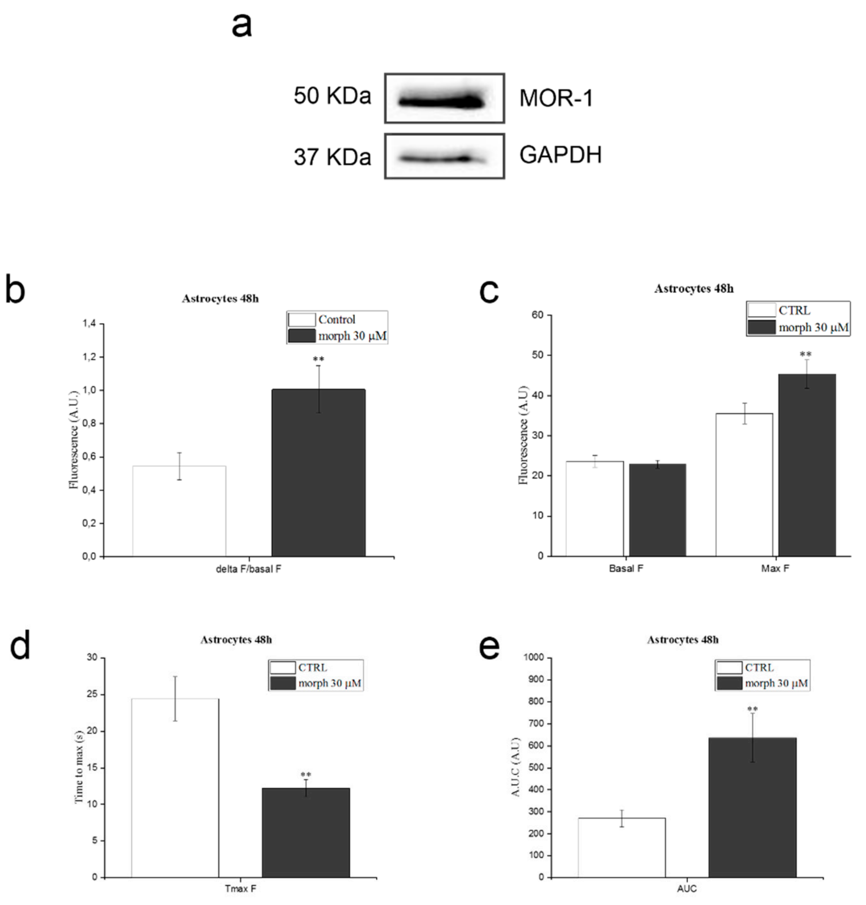

Figure S6. Effect of morphine on astrocyte calcium release. (**a**) WB of MOR-1 (μ-receptor) on rat primary cortical astrocytes, the entire blot is shown in Figure S2 (green box); (**b-c-d-e**) Intracellular calcium influx analysis on astrocytes treated for 48 hours with morphine 30 μM and stimulated with 100 μM ATP. Maximum fluorescence reached, ΔF/F, time to reach the maximum fluorescence and area under the curve (A.U.C.) were analyzed. Values are expressed as the mean ± S.E.M. of three experiments. ** $p < 0.01$ vs. control astrocytes.
